# Green Tea Consumption and Stomach Cancer Risk: A Meta-Analysis

**DOI:** 10.4178/epih/e2010001

**Published:** 2010-04-26

**Authors:** Hyunseok Kang, Sun Young Rha, Kyung Won Oh, Chung Mo Nam

**Affiliations:** 1 Department of Epidemiology and Biostatistics, Graduate School of Public Health, Yonsei University, Seoul, Korea.; 2 Department of Medicine, St.Luke's-Roosevelt Hospital Center, Columbia University College of Physicians and Surgeons, New York, NY, USA.; 3 Cancer Metastasis Research Center, Yonsei University College of Medicine, Seoul, Korea.; 4 Division of Chronic Disease Surveillance, Korea Centers for Disease Control and Prevention, Seoul, Korea.; 5 Department of Preventive Medicine, Yonsei University College of Medicine, Seoul, Korea.

**Keywords:** Green tea, Stomach cancer, Meta-analysis

## Abstract

**OBJECTIVES:**

Green tea has been suggested to have a chemopreventive effect against various cancers including stomach cancer. The aim of this study is to elucidate the relationship between green tea consumption and stomach cancer risk by meta-analysis.

**METHODS:**

Eighteen observational studies were identified using MEDLINE, THE COCHRANE LIBRARY, RISS, and a manual search. Summary relative risks/odds ratios (RR/ORs) for the highest versus non/lowest green tea consumption levels were calculated on the basis of fixed and random effect models. Subgroup analyses were used to examine heterogeneity across the studies.

**RESULTS:**

The combined results indicate a reduced risk of stomach cancer with intake of green tea (RR/OR=0.86, 95% CI=0.74-1.00). Subgroup analysis with six studies that reported differences between the highest and lowest consumption levels equal to or greater than five cups/day revealed a statistically significant protective effect (RR/OR=0.68, 95% CI=0.53-0.87).

**CONCLUSION:**

Green tea appears to play a protective role against the development of stomach cancer. The results also suggest that a higher level of green tea consumption might be needed for a clear preventive effect to appear. This conclusion, however, should be interpreted with caution because various biases can affect the results of a meta-analysis.

## INTRODUCTION

Risk factors for stomach cancer include infection with *Helicobacter pylori*, genetic factors, dietary intake, and cigarette smoking [1, 2]. Dietary intake has been suggested as an especially important factor in the etiology of stomach cancer when explaining geographic, socioeconomic, and chronologic discrepancies in the incidence [3].

Presently, tea is the most widely consumed beverage in the world aside from water [4]. Tea is generally consumed in the form of green (20%), oolong (2%), or black (78%) tea, all of which originate from the leaves of the plant *Camellia sinensis* [5]. Among the teas, green tea contains many polyphenols known as catechins, such as epigallocatechin-3 gallate (EGCG), epigallocatechin (EGC) and epicatechin-3 gallate (ECG). Tea and the constituents of tea have been shown to inhibit tumorigenesis in many animal models, including those for cancer of the skin, lung, oral cavity, esophagus, stomach, small intestine, colon, liver, pancreas, bladder, breast and prostate [6]. Mechanisms that have been proposed for the biological activities of tea polyphenols include antioxidant activities, induction or inhibition of drug metabolizing enzymes, inhibition of arachidonic acid metabolism, inhibition of cell proliferation, induction of apoptosis, and inhibition of DNA methyltransferase, dihydrofolate reductase (DHFR), protease, and telomerase [7].

Ahn et al. [8] reported significant favorable responses in women with human papilloma virus-infected cervical lesions treated with oral and/or topical green tea extract preparations, and Bettuzzi et al. [9] also reported that 600 mg of daily catechin extract derived from green tea had a statistically significant protective effect in patients with high-grade prostate intraepithelial neoplasia. Over the last three decades, a number of epidemiologic studies were conducted to investigate the association between green tea consumption and stomach cancer risk in human subjects. Recent narrative reviews have concluded that epidemiologic studies did not provide consistent evidence to support tea as a chemopreventive agent against stomach cancer development [10, 11]. There has never been any quantitative attempt, however, to summarize the results of studies exploring a possible green tea-stomach cancer association. The aim of this study is to elucidate the association between green tea consumption and stomach cancer risk by meta-analysis of previously published data.

## MATERIALS AND METHODS

### Literature search and inclusion criteria

To search for observational studies of green tea consumption in relation to stomach cancer risk, we conducted a literature search using the following medical databases, MEDLINE, THE COCHRANE LIBRARY, and RISS (to search for Korean literature); we restricted the search to papers published in English, Japanese or Korean, which were published up to May 2007. For the search, we identified articles using such medical subject-heading terms as "stomach neoplasms", "tea" or "catechin" or the keywords: "stomach cancer", "gastric cancer", "green tea", or "catechin". In addition, we also conducted a manual search of reference lists from the retrieved papers for further relevant publications.

For inclusion in the meta-analysis, the identified articles had to meet the following criteria: 1) they had to be human studies, not laboratory or animal studies; 2) they had to document the daily consumption of the natural green tea product, not of green tea extracts or supplements; 3) the outcome of interest had to be an incidence of stomach cancer; 4) fulltext articles from the study had to be accessible to the authors. We excluded studies which did not provide information on (i) the number of stomach cancer cases and controls studied and/or (ii) the odds ratio (OR) or relative risk (RR) and its corresponding 95% confidence interval (CI) for highest versus non/lowest level of tea intake. When more than one studies analyzed the same dataset, only the most recent study was included in the analysis. These articles were reviewed independently by two authors (H.K. and C.M.N.) to determine whether the articles met the inclusion criteria of the present study.

### Statistical analysis

Study-specific ORs/RRs and the corresponding 95% CIs for highest versus non/lowest green tea consumption levels were extracted from the publications. Crude OR was calculated from the numbers of cases and controls in one study [12] because the analysis included a previously published data [13]. If a study provided separate OR or RR estimates for men and women, we treated them as two different studies. For a study that provided two OR or RR estimates based on hospital and population controls, we used the estimate derived from the population control. The standard errors of the natural logarithms of the ORs or RRs were calculated from the 95% CIs of the ORs/RRs and used for the meta-analysis. Statistical computing was performed using the STATA statistical software (version 8.0; College Station, TX, USA).

Possible heterogeneity of effect sizes across the studies was examined using the Q statistic [14]. Statistical significance for the heterogeneity test was defined as p<0.10 rather than the conventional level of 0.05 because of the low power of this test [15]. When there was significant heterogeneity among effect sizes, the random effect model was used to calculate the summary RR/OR. When the results of the studies were homogenous, the fixed effect model was used instead. The causes of heterogeneity were further explored through subgroup analyses.

For calculation of the difference between the highest and lowest consumption levels of green tea, all the measured consumption levels were converted to a cups-per-day scale. Each gram of green tea consumed shown in two of the Chinese studies was converted to 0.25 cup following the suggestion by Mu et al. [11]. For the study by Nagano et al. [16], we assumed one cup of green tea would be consumed at a time. Yu et al. [17] reported the number of new batches of green tea used. Among those who used more than four batches a day, 12% brewed 1-3 cups per batch and 88% brewed more than four cups per batch. The number of cups per day consumed was calculated using the following equation: (0.12×2+0.88×4)×4=15.

To detect a possible publication bias, Begg's funnel plot was visually evaluated for any asymmetry. To formally test for a publication bias, Egger's un-weighted regression asymmetry test was done [18]. The funnel plot was considered to be asymmetrical if the intercept of Egger's regression line deviated from zero with a p value of less than 0.05.

## RESULTS

**Figure 1:**
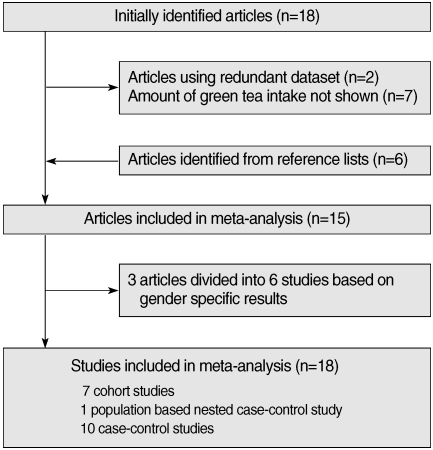
Flow of study selection process.

Eighteen articles [11-13, 17, 19-32] were identified in the initial search with the above mentioned method. Six articles [16, 33-37] were identified from reference lists. Two studies [24, 34] using a redundant dataset were excluded. Seven other articles [12, 20, 26, 29-32] were excluded since they lacked information on the amount of green tea intake. As a result, fifteen articles were found to meet the inclusion criteria described above [11-13, 16, 17, 19, 22, 23, 27, 28, 33-37]. As three articles having gender-specific results were divided into six independent studies [22, 28, 33], a total of 18 studies were included in the study in the end. The selection process is shown in Figure 1. There were seven cohort studies, one population-based nested case-control study, and ten case-control studies. Twelve studies were conducted among the Japanese population in Japan, five studies were conducted among the Chinese population in China, and the other was conducted among the Japanese-born population in Hawaii, USA. All of the Chinese studies were case-control studies. Table 1 presents the characteristics of the studies used in the analysis.

**Figure 2:**
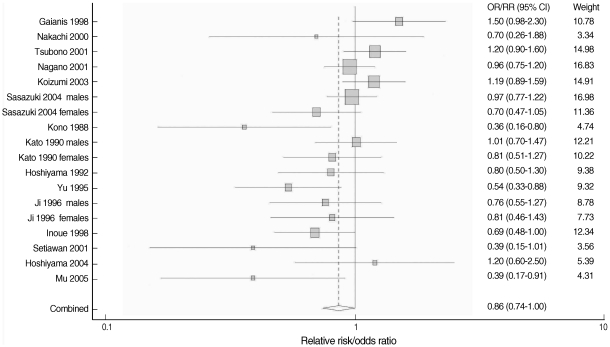
Forest plot of relative risks or odds ratios from eighteen observational studies on green tea consumption and stomach cancer. The black squares and horizontal lines correspond to the RRs or ORs and 95% confidence intervals. The area of the black squares reflects the weight each trial contributes to the meta-analysis. The diamond at the bottom of the graph represents the combined odds ratio and its 95% confidence interval, indicating 14% reduction in the risk of stomach cancer. The solid vertical line corresponds to no effect of green tea consumption (odds ratio 1.0), the dotted vertical line to the combined odds ratio (0.86). The graph was produced in STATA.

The overall result, which is presented in Figure 2, showed a statistically significant, 14% reduction in the risk of stomach cancer with high green tea consumption (summary RR/OR=0.86, 95% CI=0.74-1.00). Table 2 presents the results of the subgroup analyses. When stratified by country (Japan versus China), results were homogenous among five Chinese studies (p=0.43) with a significant risk reduction of 39% (summary RR/OR=0.61, 95% CI=0.47-0.81). Results from the twelve Japanese studies were marginally homogenous (p=0.10) with a non-significant risk reduction of 8% (summary RR/OR=0.92, 95% CI=0.80-1.05). A statistically significant inverse association between green tea intake and stomach cancer was observed only in the eleven case-control studies (summary RR/OR=0.74, 95% CI=0.63-0.86). Results from the seven cohort studies failed to support the association (summary RR/OR=1.03, 95% CI=0.92-1.16).

When stratified by gender, the results among men were divergent (p=0.02) while the results among women were consistent (p=0.53). Neither the studies of men nor of women showed any significant reduction in the risk (summary RR/OR=1.00 and 0.89, 95% CI=0.82-1.24 and 0.74-1.07, respectively). When stratified by difference between the highest and lowest green tea consumption levels, results among six studies with the difference equal to or greater than five cups/day were homogeneous (p=0.30) with a statistically significant risk reduction of 32% (summary RR/OR=0.68, 95% CI=0.54-0.85). Twelve studies with a difference of less than five cups/day showed heterogeneous results (p=0.04) with a non-significant risk reduction of 6% (summary RR/OR=0.94, 95% CI=0.81-1.10).

**Figure 3:**
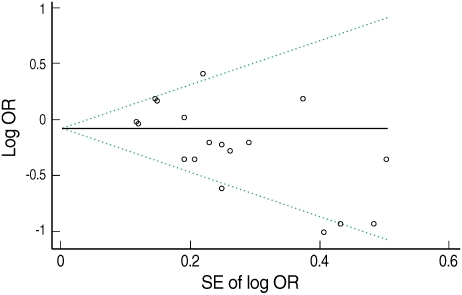
Begg's funnel plot of studies on green tea consumption and stomach cancer risk. The solid line in the center is the natural logarithm of pooled odds ratio (OR), and two oblique lines are pseudo 95% confidence limits. SE, standard error.

Figure 3 presents Begg's funnel plot. Visual exploration of the plot revealed an apparent asymmetry-smaller studies tended to report a protective effect of green tea while larger studies showed more mixed results. The result of Egger's test also supported the suspicion of publication bias (intercept=-2.02, p=0.01).

## DISCUSSION

This meta-analysis investigated the association between green tea consumption and stomach cancer risk on the basis of previously published researches. The overall summary RR/OR for green tea consumption and stomach cancer risk, as derived from eighteen observational studies, indicated a statistically significant 14% risk reduction in the high green tea consumption group.

The reduced risk of stomach cancer in green tea drinkers was observed in studies with differences between the highest and lowest daily green tea consumption levels equal to or greater than five cups per day. Also, the studies conducted in China showed a stronger reduction in stomach cancer among green tea drinkers than those conducted in Japan. A few authors have argued that the relative lack of subjects in Japan who do not drink green tea may have resulted in an insufficient number of non-drinkers, and this might be an explanation for the weaker associations among Japanese studies [11, 16, 38]. When a meta-analysis was done with the four Japanese studies in which the difference between the highest and lowest consumption levels was greater than five cups/day [19, 27, 34, 37], the summary RR/OR and 95% CI were 0.72 and 0.53-0.97. This implies that if a large prospective study with a more detailed categorization of green tea consumption were performed, a protective effect of large amount of green tea intake might be shown. This also implies that the lack of protective effect of green tea shown in studies which compared relatively lower level of green tea consumption might be because of not enough intake of green tea itself. Another possible explanation is the difference in the production processes among Japan and the other countries. In Japan, green tea production involves a steaming process at a high temperature to retain the green color of the tea. This process may lead to changes in chemical composition and in the concentrations of bioactive constituents such as vitamins C and E, which may also contribute to the chemopreventive properties of green tea [11]. Also, the bioactivity of a cup of green tea differs by the amount of green tea leaves used to brew it and the frequency of renewing a tea batch in the pot [16]. Differences in tea preparation and drinking habits may, therefore, be a partial explanation for the differing results.

Research design also seemed to play an important role in the heterogeneity of effect sizes across the studies. While the protective effect was observed among case-control studies only, prospective studies tended to show null results. A few prospective studies even showed increased risks with green tea consumption although they were not statistically significant [12, 13, 35]. Some authors suggested that tea might have a mutagenic effect [35], but this hypothesis is contradictory to the results of most laboratory research. The number of cases was very small for the green tea drinkers in the report by Galanis et al. [35], and that may have resulted in the exaggerated risk estimates.

Sasazuki et al. [28] suggested that there might be a gender-specific protective effect of green tea on stomach cancer, but the analysis of seven studies that included gender information revealed a statistically non-significant effect on both genders (estimates of RR/OR=1.00 for men and 0.89 for women, 95% CI=0.82-1.24 for men, 0.74-1.07 for women). Thus, gender does not seem to cause any difference in the effect of green tea on stomach cancer risk.

Site-specific stomach cancer incidence in accordance with green tea consumption was mentioned in four studies [12, 17, 22, 28]. While Ji et al. [22] and Koizumi et al. [12] showed no difference between green tea consumption and stomach cancer risk by anatomical subsite(data not shown), the other two authors reported a different risk pattern by subsite. Yu et al. [17] showed a significant protective effect for pyloric tumors (OR= 0.29, 95% CI=0.13-0.68), and Sasazuki et al. [28] reported a significant effect for distal tumors among women (OR=0.53, 95% CI=0.30-0.86). Although more studies are needed to address this issue, it is possible that green tea consumption might be related to distal stomach cancers only. It is recommended that future studies take this into consideration.

Total duration of green tea drinking was considered in three of the Chinese studies [11, 17, 23]. All of them showed a decreased risk of stomach cancer with increasing duration of green tea drinking, but this result failed to reach statistical significance (data not shown). Further studies are needed to clarify this point. It is known that *Helicobacter pylori* infection is an important risk factor for a stomach cancer [1]. Only two studies [11, 23], however, controlled for the bacteria infection in their analyses. Green tea has been considered to have bacteriostatic and bactericidal effects [39, 40], which can extend to *Helicobacter pylori*. Thus, the infection could have confounded the results, and further study is needed.

This meta-analysis has several limitations. First of all, publication bias cannot be ruled out. As shown in the subgroup analysis, the protective effect of green tea was prominent among case-control studies, which could be easily misled by a publication bias. Because of the asymmetric funnel plot, publication bias cannot be ruled out in this study. Second, the research included in this study had different categories for green tea consumption. Although the odds ratio or relative risk of the highest consumption versus non/lowest consumption was used for combining the effect size, it was not uniform across the studies. This might have distorted the result. Third, the (non-English) Chinese literature could not be reviewed because of the language barrier. Because results from the Chinese studies tended to show protective effects, the combined effect would have been different if they had been included in the study. Last, all of the studies included in the analysis had been done among Asian populations. Green tea is a popular drink in East Asia, while black tea is mostly consumed in Western countries. The result of this study cannot be applied to non-Asian populations.

In summary, the result of this meta-analysis suggests a protective role of green tea against stomach cancer. Subgroup analyses revealed that the difference between the measured highest and lowest green tea consumption level was found to be the most prominent factor affecting the heterogeneity of the meta-analysis. This implies that the daily consumption level might be an important factor in determining the preventive effect of green tea against stomach cancer. Further research focusing on higher green tea consumption level is needed to clarify the association.

## Figures and Tables

**Table 1:**
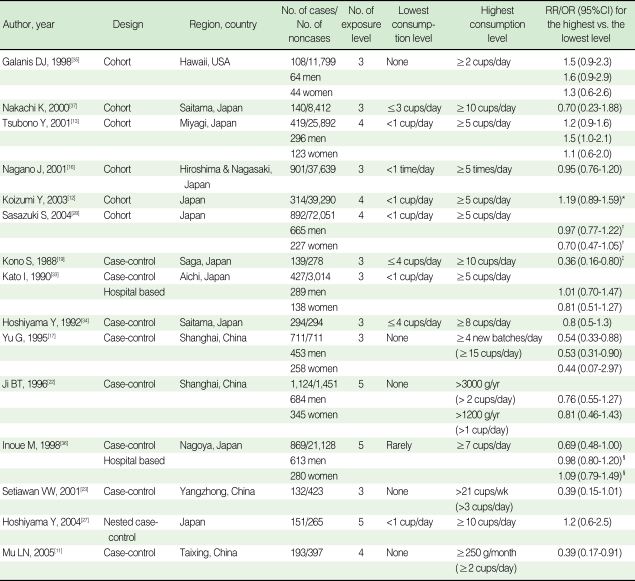
Characteristics of studies on green tea consumption and stomach cancer risk

Case-control studies are population-based unless otherwise specified. ^*^In two cohorts reported in the article, crude RR and 95%CI were calculated from the numbers from the previously unpublished cohort 2; ^†^RR/ORs were the results of a pooled analysis of two cohorts reported in the study; ^‡^Crude RR/OR and 95%CI were calculated from the numbers shown in the study; ^§^Crude RR/ORs and 95%CIs were calculated from the numbers given in the study for men and women respectively.

**Table 2:**
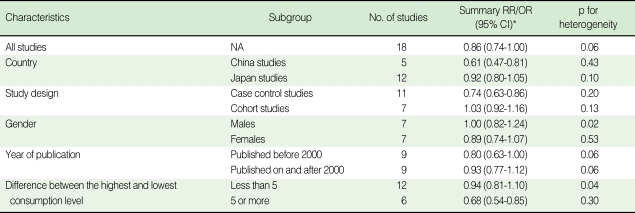
Subgroup analysis of green tea consumption and stomach cancer risk

^*^Estimates of the summary RR/ORs and 95% CIs were based on either random effect model if the studies included are heterogeneous (i.e. p for heterogeneity is less than 0.10), or fixed effect model if the studies included are homogenous.
